# S14. DNA METHYLATION CHANGES IN GABAERGIC AND GLUTAMATERGIC MARKERS IN EARLY SCHIZOPHRENIA

**DOI:** 10.1093/schbul/sby018.801

**Published:** 2018-04-01

**Authors:** Helene Fachim, Camila Loureiro, Fabiana Corsi-Zueli, Paulo Rossi Menezes, Paulo Louzada, Caroline Dalton, Cristina Marta Del-Ben, Gavin Reynolds

**Affiliations:** 1 Sheffield Hallam University; 2 University of Sao Paulo

## Abstract

**Background:**

GABAergic and glutamatergic systems play an important role in the neurobiology of schizophrenia, and changes in their markers are reported in both postmortem human brain and in animal models. Recent studies have demonstrated that abnormalities in DNA methylation may underlie the alterations in various indicators of GABAergic and glutamatergic functions in schizophrenia. As our group previously found decreased NR2 protein plasma levels and downregulation of parvalbumin (PVALB) mRNA in first episode of psychosis (FEP) patients, we hypothesised that changes in DNA methylation may be responsible for these indicators of glutamatergic and GABAergic deficits in FEP patients.

**Methods:**

Blood samples were collected from patients in FEP (n = 35) after their first contact with the mental health assistance, siblings (n = 21) and population-based controls (n = 35). Bisulfite conversion and pyrosequencing were used to determine methylation levels in 4 CpG sites in promoter sequence of PVALB and 5 CpG sites at GRIN2B (gene which encodes NR2).

**Results:**

We found hypermethylation at a CpG site within the PVALB promoter sequence in patients and their siblings compared to population-based control group (p< 0.001) while overall hypomethylation was found in the 5 CpGs analysed within GRIN2B promoter sequence (p < 0.01).

**Discussion:**

Our PVALB findings are consistent with our previous studies showing that PVALB promoter methylation is elevated in schizophrenia and, additionally this is the first evidence showing changes in GRIN2B promoter methylation in psychosis. These results together suggest that these epigenetic findings may relate to the reduction of protein expression of indicators of glutamate and GABA systems seen in this disease.

